# Genome-Wide Association Mapping in Dogs Enables Identification of the Homeobox Gene, *NKX2-8*, as a Genetic Component of Neural Tube Defects in Humans

**DOI:** 10.1371/journal.pgen.1003646

**Published:** 2013-07-18

**Authors:** Noa Safra, Alexander G. Bassuk, Polly J. Ferguson, Miriam Aguilar, Rochelle L. Coulson, Nicholas Thomas, Peta L. Hitchens, Peter J. Dickinson, Karen M. Vernau, Zena T. Wolf, Danika L. Bannasch

**Affiliations:** 1 Department of Population Health and Reproduction, School of Veterinary Medicine, University of California Davis, Davis, California, United States of America; 2 Department of Pediatrics, University of Iowa, Iowa City, Iowa, United States of America; 3 Medical Microbiology and Immunology, Genome Center, MIND Institute, University of California Davis, Davis, California, United States of America; 4 Department of Plant Pathology, University of California Davis, Davis, California, United States of America; 5 JD Wheat Veterinary Orthopedic Research Laboratory, School of Veterinary Medicine, University of California Davis, Davis, California, United States of America; 6 Department of Surgical and Radiological Sciences, School of Veterinary Medicine, University of California Davis, Davis, California, United States of America; University of Bern, Switzerland

## Abstract

Neural tube defects (NTDs) is a general term for central nervous system malformations secondary to a failure of closure or development of the neural tube. The resulting pathologies may involve the brain, spinal cord and/or vertebral column, in addition to associated structures such as soft tissue or skin. The condition is reported among the more common birth defects in humans, leading to significant infant morbidity and mortality. The etiology remains poorly understood but genetic, nutritional, environmental factors, or a combination of these, are known to play a role in the development of NTDs. The variable conditions associated with NTDs occur naturally in dogs, and have been previously reported in the Weimaraner breed. Taking advantage of the strong linkage-disequilibrium within dog breeds we performed genome-wide association analysis and mapped a genomic region for spinal dysraphism, a presumed NTD, using 4 affected and 96 unaffected Weimaraners. The associated region on canine chromosome 8 (*p_genome_* = 3.0×10^−5^), after 100,000 permutations, encodes 18 genes, including *NKX2-8*, a homeobox gene which is expressed in the developing neural tube. Sequencing *NKX2-8* in affected Weimaraners revealed a G to AA frameshift mutation within exon 2 of the gene, resulting in a premature stop codon that is predicted to produce a truncated protein. The exons of *NKX2-8* were sequenced in human patients with spina bifida and rare variants (rs61755040 and rs10135525) were found to be significantly over-represented (p = 0.036). This is the first documentation of a potential role for *NKX2-8* in the etiology of NTDs, made possible by investigating the molecular basis of naturally occurring mutations in dogs.

## Author Summary

Neural tube defects (NTDs) are birth defects resulting from errors in the closure of the neural tube, an embryonic structure which develops into tissues of the central nervous system during pregnancy. NTDs commonly lead to costly lifelong disabilities. They are considered to be caused by a combination of nutritional, inherited and environmental factors, and their interactions. However, an obvious mechanism is currently unknown. Genetic studies in human populations are made difficult by the multifactorial nature of NTDs and because multiple cases within a single family are rare. Animal models are helpful in dissecting the genetics of such complex traits; however existing rodent models do not explain all of the NTD cases in humans. Dogs are excellent biomedical models for humans since they receive comparable medical care, share our home environment, and develop naturally occurring diseases comparable to those in humans. We used a naturally occurring NTD in Weimaraner dogs, termed spinal dysraphism, to identify a mutation in an associated regulatory gene, *NKX2-8*. Mutations in *NKX2-*8 were subsequently documented in human patients with a generally similar NTD termed spina bifida. This is the first documented evidence that *NKX2-*8 has a role in NTDs. It is expected that this discovery will contribute to our understanding of the mechanisms leading to NTDs.

## Introduction

Neural tube defects (NTDs) is a general term used to describe developmental defects resulting from abnormal closure or development of the neural tube during embryogenesis. The resulting pathologies may involve the brain, spinal cord, and associated structures such as vertebrae, soft tissue or skin [1]. The condition is reported among the more common birth defects in humans (incidence of ∼1 per 1,000 pregnancies worldwide), leading to significant infant morbidity and mortality [2], [3]. Clinically, human NTDs are defined as a severe, “open” form, in which tissues of the nervous system are exposed to the environment or a “closed,” form, characterized by skin-covered lesions [1]. NTDs are the outcome of aberrant primary or secondary neurulation during embryogenesis. During primary neurulation, the neural plate folds on itself and fuses on the midline into the neural tube [4]. The brain and most of the spinal cord are formed by primary neurulation. Flawed primary neurulation typically leads to open forms of NTDs which include anencephaly and spina bifida. Spina bifida (failure of vertebral fusion) usually occurs secondary to failure of closure of the neural tube, and causes a spectrum of physical and developmental disabilities, depending on the magnitude and position of the spinal defect [3]. Spinal dysraphism is an alternative terminology, describing conditions with malformations of structures relating to the midline raphe of the developing spine; generally implying neural tube defects [5]. Secondary neurulation is defined as the formation of the caudal portion of the neural tube from the pluripotent cells of the tail bud and does not require folding as the solid cell mass undergoes cavitation. Secondary neurulation creates most of the sacral and all of the coccygeal tissues of the spinal cord [4]. Pathologic secondary neurulation may result in closed forms of spina bifida, where the nervous tissue fails to separate from the other tissues of the tail bud [6].

The etiology of NTDs remains poorly understood and genetic, nutritional (folate, inositol), and environmental factors, or a combination of these, are known to play a role in the development of NTDs [1], [2]. Genes involved in the complex multistep process of neurulation such as those in the the planar cell polarity (PCP) pathway [4], [7], and genes involved in folate metabolism [8], [9], have been found to contribute to NTDs. Mouse models have led to the identification of over 200 genes with roles in NTDs [10], [11]. In addition to null mutants, many knock-down or compound mutants have been identified, and some mutations, like the kinky tail mouse [12]–[14], cause NTDs in oligogenic combinations, or like the curly tail mouse, display complex inheritance [11], [15], [16].

Similarly, it is presumed that most human NTDs have a multifactorial etiology and arise in a similar fashion to some of the mouse phenotypes [6]. Although multiple genetic variants that increase the risk of developing NTDs have also been recognized for humans [17], the identified genetic variation does not explain the total genetic contribution to the incidence of NTDs observed in human populations [3]. Utilizing conventional approaches such as positional cloning and genetic linkage to identify additional associated variants is hampered by the rarity of families with multiple affected individuals, and because of undersized cohorts leading to suboptimal power for association studies [3], [17].

In contrast to association studies in human populations, the dog is a large animal model that is particularly useful for whole genome association studies due to its unique population structure [18], [19]. Haplotype blocks in LD (linkage disequilibrium, non-random association of alleles at two loci or more) extend across 0.4 to 3.2 megabases [20], [21], and are similar to the extent of LD in inbred strains of mice; simplifying genetic analyses [22], [23]. In addition, the variable conditions associated with NTDs occur naturally in dogs [24], and spina bifida has been reported to occur sporadically in breeds such as the English Bulldog [25], Toy Poodle [26], [27] Collie, Chihuahua, mixed-bred dogs [27] and Samoyed [28]. Furthermore, Weimaraner dogs with a NTD, commonly referred to as “spinal dysraphism” were studied previously [29], [30]. Research colonies of affected Weimaraners have been maintained [31], [32], and breeding experiments included mating of severely affected Weimaraner dogs which resulted in 10/10 affected fetuses [32], [33], suggesting a recessive mode of inheritance. However, other breeding experiments did not support a conclusive mode of transmission [30] and the disorder was speculated to have a complex mode of inheritance in the Weimaraner [29].

Extensive studies by McGrath [30], demonstrated that the heterogenic spinal pathology in Weimaraners includes duplicated, stenotic, or absent central canal, hydromyelia or syringomyelia, chromatolysis and loss of nerve cell bodies in gray matter, disrupted dorsal median septum and ventral median fissure, and gray matter ectopias [30]. In any affected Weimaraner, these histopathological changes may be present in varying degrees within different spinal cord segments, but occur most frequently in the lumbosacral region. Engel and Draper [33] reported that abnormalities in affected Weimaraner prenates were evident in embryos (24 days of gestation) and consisted of failure of the dura mater to separate from the periosteum, absence of the ventral median fissure and fusion of ventral white matter, and disruption of gray matter structure. Central canal diverticula were common and the diameter ratio of gray matter/spinal cord was significantly greater in affected fetuses [33].

In the live Weimaraner, McGrath [30] observed abnormal hair streams along the back, similar to those observed in some of the human patients [34], kinked tails resembling the curly and kinked tail mouse phenotypes [15], [12], and scoliosis of the vertebral column in the lumbar spinal region [30]. Clinical signs include paraparesis and a symmetric “bunny-hopping” or simultaneous use of the pelvic limbs, a bilateral withdrawal reflex; pinching one paw elicits flexion of both hindlimbs, a crouched stance, and deficient proprioception in the pelvic limbs [30]. Although spina bifida was not observed in Weimaraner cases, they share many of the human and mouse NTDs phenotypes which suggests that an evolutionary conserved molecular pathway may be contributing to the pathoetiology of NTDs across these species.

Whereas previously maintained colonies of affected Weimaraners allowed for a thorough description of the phenotype and experimental breeding has confirmed that “spinal dysraphism” is an inherited condition in the Weimaraner breed, no mutation has been identified to date. We used four cases of presumed spinal dysraphism to map a genomic location for the disorder in the breed. A regional homeobox candidate gene with functions in neuronal development, *NKX2-8*, was sequenced and a frameshift mutation was identified in affected Weimaraners. Human patients with spina bifida had a significant increase in the rate of rare missense mutations within evolutionary conserved residues of *NKX2-8*.

## Results

**Figure 1:**
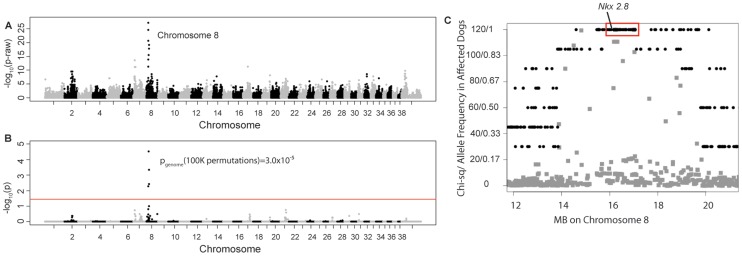
Manhattan plots of GWAS results for NTDs in Weimaraners (4 cases, 96 controls; λ = 1.03). **A**. Raw p-values. Y axis: −log 10 of the raw p-values; X axis: SNPs color coded by chromosome. The lowest p-values are on chromosome 8. **B**. 100K Max (T) permutation results. Y axis: −log 10 of the permuted p-value; X axis: SNPs color coded by chromosome. The red line denotes genome wide significance (p≤0.05; −log 10≥1.3). **C**. Chi-square and allele frequencies for affected dogs by Mb on chromosome 8. The interval with the highest chi-square association (χ^2^ = 119) and allele frequency = 1 within affected dogs is boxed, defining the critical interval. *NKX2-8* is located within this interval.

Four unrelated Weimaraners showing the clinical signs typical of “spinal dysraphism” (Video S1, Figure S1) and 96 unaffected Weimaraners were genotyped using ∼173 k SNP markers. Following quality control, 114,775 SNPs were retained in the genome-wide association study (GWAS) analysis using PLINK, and an associated region on canine chromosome 8 was observed (Figure 1A and 1B). To confirm that this region was not falsely associated due to population substructure, we reviewed the quantile–quantile plots with the associated SNPs on chromosome 8 (λ = 1.03, Figure S2A) and without the SNPs on chromosome 8 (λ = 1.01, Figure S2B). The associated region extended over ∼1.5 Mb. Within this region, a distinct homozygous haplotype was present within the affected dogs (Figure 1C), and was absent in all 96 unaffected dogs.

**Figure 2:**
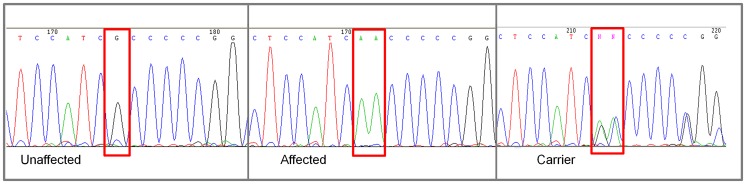
Chromatograms of *NKX2-8* exon 2 sequence where a mutation of G to AA was found in an affected Weimaraner. From left to right: Unaffected Weimaraners have a genotype of “GG”; affected Weimaraners have a genotype of “AA”; carrier Weimaraners have a genotype of “AG”.

Of the 18 regional genes (Table S1) *NKX2-8* (chr8: 18,156,525–18,157,928) was shown to regulate key steps in spinal accessory motor neuron development in the mouse, and adult mice with targeted disruptive *NKX2-8* mutations exhibit abnormal locomotion, including a permanent or intermittent hopping gait. The two exons of *NKX2-8* were sequenced in genomic DNA and an alteration of a G to AA was identified within exon 2 in an affected Weimaraner when compared to unaffected Weimaraners and to the Boxer reference genome [21]. Two obligate carriers (parents of affected dogs) and two littermates of affected dogs were heterozygous for the mutation. The three genotypic variants observed within exon 2 of *NKX2-8* are shown in Figure 2.

**Figure 3:**
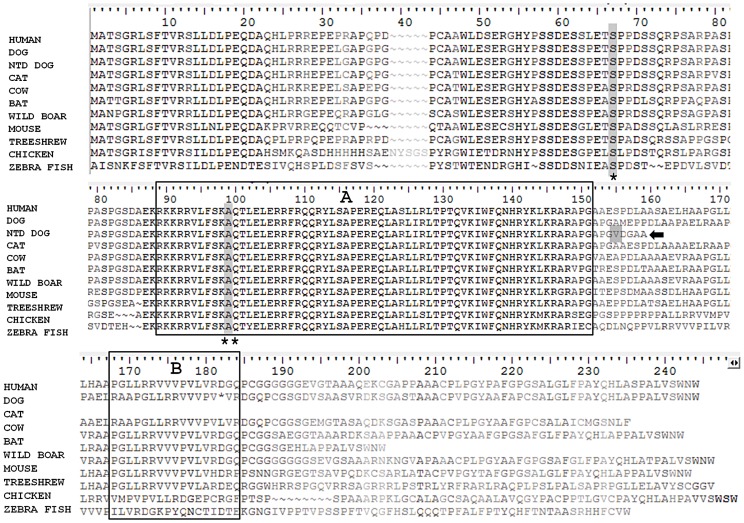
Comparison of the protein sequence of *NKX2-8* between human, unaffected dog, spinal dysraphism Weimaraner, cat, cow, bat, wild boar, mouse, tree-shrew, chicken and zebra fish. Two functional domains: a homeobox (A) and an NK specific domain (B) are boxed. A truncated protein (arrow), the result of the frameshift mutation (grey shaded) in spinal dysraphism affected Weimaraners, is missing the NK specific domain. Locations for missense variants rs61755040 (asterisk) and rs10135525 (double asterisk), found in human patients with spina bifida, are shaded within the protein sequence. These variants reside within evolutionary conserved domains.

In order to determine the *NKX2-8* protein sequence, we acquired the complete cDNA sequence, including the 5′ and 3′ untranslated regions from brain tissue of an unaffected Beagle. Subsequent translation of the exonic sequence of an affected Weimaraner revealed that the identified alteration functions as a frameshift mutation which introduces an amino acid change (*A150VfsX1*) and a downstream stop codon (Figure 3). No additional mutations were identified within the promoter region or within the exon-intron boundary of *NKX2-8* in genomic DNA of affected dogs. 109 additional unrelated unaffected Weimaraners were tested for the presence of the *A150fs* mutation by direct sequencing and three carrier dogs were identified. The mutation frequency was therefore calculated to be ∼1.4% within the Weimaraner breed. One additional case of clinically affected Weimaraner had no copies of the mutation. Additionally, 496 unaffected dogs, from six breeds reported to be clinically affected by NTDs, and a Chesapeake Bay Retriever diagnosed with myelodysplasia, absent ventral median fissure, hydromyelia, and syringomyelia by histopathology, were tested to determine whether or not this is an allelic mutation. No copies of the mutation were found within non-Weimaraner dogs.

To investigate a potential role for *NKX2-8* in cases of NTDs in human patients, 149 unrelated samples from patients with lumbosacral myelomeningocele, (spina bifida), were sequenced. Six missense variants were identified in exon 2 of *NKX2-8* within the spina bifida cohort. Five patients (3 females, 2 males), all European Americans were heterozygous for variant rs61755040, which has a reported minor allele frequency (MAF) of 0.0073 in dbSNP. This missense variant, results in an amino acid change of serine to threonine at position 62 of human *NKX2-8*, within an area of complete evolutionary conservation (Figure 3). The *S62T* alteration is predicted to be “probably damaging” by PolyPhen. Of the 149 samples, only 19 belonged to African American spina bifida patients. While we did not have adequate sample size to examine this ethnic group statistically, we did find that one female African American was heterozygous for variant rs10135525, which has a reported MAF of 0.0014 in dbSNP. This missense mutation results in an amino acid change of alanine to threonine at position 94, within the evolutionary conserved homeobox functional domain of human *NKX2-8* (Figure 3) and is also predicted to be “probably damaging” by PolyPhen. As shown in Figure 3, both variants reside within domains of 100% identity between human (Homo sapiens), dog (Canis lupus familiaris), cat (Felis catus), cow (Bos taurus), bat (Pteropus alecto), wild boar (Sus scrofa), mouse (Mus musculus), tree-shrew (Tupaia chinensis), chicken (Gallus gallus) and zebra fish (Danio rerio).

Using the Exome Variant Server (EVS) data as a control population for spina bifida, we compared missense variants in the European American spina bifida population versus the EVS population. The EVS European American database contains 6 variants in *NKX2-8* (nonsense or missense) in a total of 72 variant alleles out of an average of 8,500 alleles sequenced (Table S2). The difference between the frequency of missense variants in spina bifida cases versus controls was significant by one tailed Chi-squared analysis with Yate's correction (p = 0.036).

## Discussion

Using LD mapping in dogs, we identified an *A150fs* frameshift mutation which segregates within the Weimaraner breed in spinal dysraphism affected dogs and their relatives. The frameshift mutation was absent in 496 dogs from six breeds that were previously reported in the literature as presenting with cases of spina bifida, and in a case of spinal dysraphism in a Chesapeake Bay Retriever dog. Our results suggest that this is a private mutation in Weimaraners which is not shared between breeds. The mutation does not segregate as a benign polymorphism in canine populations, supportive of a causative role for the mutation.

The mutation was found in a homozygous state within spinal dysraphism cases and recessive Mendelian transmission was verified by genotyping two parents (obligate carriers) and two littermates whose samples were available. Additionally, the ∼1.5 Mb genome-wide associated region is comprised of a homozygous haplotype present in the affected dogs which best fit a recessive model of inheritance [35]. Previously, Karlsson et al. demonstrated that a Mendelian recessive trait could be successfully mapped within a single dog breed with fewer than 15 cases and 15 control dogs [18]. We used a genome-wide case-control association study to map spinal dysraphism with merely four cases; providing further evidence for the effectiveness of the dog as a model organism for inherited diseases.

The ∼1.5 Mb associated haplotype contained a tight cluster of associated SNPs and 18 regional candidate genes. Among these genes, *NKX2-8* was an appealing candidate since it belongs to a family of vertebrate developmental regulators (homeodomain transcription factors) that are homologues of the Drosophila homeodomain transcription factor, *NK2* [36]–[38]. *NKX2-8* is expressed in the developing neural tube [39], connecting it with the sub-group of the *Nk2* genes which is expressed in the central nervous system [36], [37]. This group includes the Drosophila *vnd* gene and the vertebrate *Nkx2.1* and *Nkx2.2* genes [39]. This suggests an early role for the *Nk2* gene family in the development of the nervous system before the divergence of deuterostomes. The *NKX2-8* protein has an N-terminus conserved homeobox DNA binding domain; involved in the transcriptional regulation of key developmental processes, and a C-terminus conserved NK specific domain with transcriptional activity [40]. The *A150fs* frameshift mutation identified in Weimaraners introduces an early stop codon and the truncated protein lacks the NK specific domain. This mutation may lead to impaired *NKX2-8* function during embryonic development and thus the observed neurospinal pathologies in homozygous Weimaraners. Support for this proposed etiology come from extensive experiments in mice [41].

The function of the murine *NKX2-8* homolog, *Nkx2-9*, was evaluated by targeted disruptive mutations in the related homeodomain transcription factor *Nkx2-2* [42], and in *Nkx2-9* [43]. The experiments suggested that both proteins play essential and partially redundant roles in the development of distinct neuronal populations in hindbrain and ventral spinal cord [41]. The authors observed impaired floor plates that led to defects in axonal pathfinding of commissural neurons in *Nkx2-9* mutants. Intriguingly, adult mice with disruptive mutations in *Nkx2-9* exhibit varying degrees of abnormal locomotion, observed predominantly for hindlimbs as continuous hopping with no alternating activity of left and right legs, similar to the Weimaraner phenotype (supplementary video) [41]. In vitro recordings in spinal cord preparations from newborn mutant mice, showed markedly reduced coordination of locomotor-like activity with increased variability in both left-right and flexor-extensor coordination [41]. The authors concluded that disruption of the *Nkx2-9* gene results in a strong walking impairment and substantial locomotor deficits, both in vitro and in vivo [41]. Interestingly, only 75–90% of homozygous *Nkx2-9* mutants exhibit the hopping gait phenotype, suggesting that there is reduced penetrance in the mouse, or possibly a “leakage” of the null phenotype [44]. While spinal dysraphism in Weimaraners was previously reported as a disorder for which the penetrance is reduced [30], we have not seen evidence for reduced penetrance within the tested group of Weimaraners (n = 210).

A single case of a Weimaraner with clinical signs of ataxia and paraparesis did not share the *NKX2-8 A150fs* mutation. It is possible that a second mutation exists in Weimaraners, which may account for the previously reported inconsistent transmission [30]. It also cannot be ruled out that in this case the clinical signs are the result of environmental factors such as maternal hyperthermia, nutritional imbalances, medication or abnormal glucose metabolism; factors mentioned in epidemiological studies as leading to congenital spinal defects [1]. In absence of diagnostic imaging or histopathological evidence, only a suggestive diagnosis could be made for spinal dysraphism based on case history, signalment and findings on a neurological examination. The identification of a mutation which segregates in the breed may aid in reaching a diagnosis in live pet dogs. In the future, Weimaraner breeders will be able to select against this mutation through DNA screening of prospective breeding animals. Identification of a mutation leading to a NTD in the dog could be utilized to improve our understanding of NTDs in human patients.

When a cohort of 149 spina bifida patients was tested, we identified 6 cases that had one of two heterozygous missense mutations (rs61755040 or rs10135525) within exon 2 of the *NKX2-8* gene. Interestingly, the dog frameshift mutation is also located in exon 2 of the *NKX2-8* gene, suggesting that exon 2 might be more susceptible to damaging DNA mutations than exon 1 of *NKX2-8*. The missense mutations identified in spina bifida patients alter evolutionary conserved amino acid residues and functional consequences are predicted. While these variants are rare (MAF<0.007) in controls, they may explain 4% of the cases within this cohort. Nonetheless, future functional studies are needed in order to confirm that these are harmful mutations which may cause a mutant phenotype under certain conditions. Previous studies that present existing evidence to support a causative role for the variants identified within *NKX2-8* include the work performed on *VANGL1*, one of the genes of the well-studied PCP pathway [45], [46]. Similar to our results; *Loop-tail* (*Lp*) mice with NTDs had recessively inherited mutations, but when human patients were sequenced, heterozygous missense mutations were identified within the *VANGL1* gene. This suggests that heterozygous missense mutations within genes with critical functions during development play a role in the etiology of NTDs in human patients. Another discovery of heterozygous variants in human patients was made while investigating the *FZD6* gene of the PCP pathway [47]. While the inheritance in the mouse model was recessive, the variants discovered within human patients were heterozygous [48], resembling our results.

It is possible that the variants identified in spina bifida patients, have a dominant negative effect. *NKX2-8* has a DNA-binding homeobox domain which is shared by a large variety of transcriptional regulators involved in controlling development [49]. The mutations in spina bifida patients were identified in domains of absolute evolutionary conservation; both adjacent to, and within the homeobox domain. Missense mutations within homeobox domains of various genes were studied previously to reveal that in patients with congenital diseases, most missense mutations have dominant effects [49]. Amino acids of homeobox domains play the critical roles of determining the correct structural fold of proteins, regulating DNA-protein interactions, regulating protein-protein interactions and signaling nuclear localization. It was previously proposed that heterozygous mutations within homeobox genes may have a detrimental effect during development due to haploinsufficiency [50], [51].

While it is possible that the mutations identified in *NKX2-8* have a dominant negative effect, it was previously hypothesized that NTDs inheritance is multifactorial [1]. A threshold model is used to explain multifactorial contribution to the NTD dichotomous phenotype, where multiple genetic variants interact with environmental factors to cause NTDs [52]. According to the threshold model, the variants contributing to the elevated risk would be present in controls, but a significant higher frequency of these variants is expected in cases [52], [53]. Our results are consistent with this theory; however, further studies are required in order to determine the inheritance pattern of *NKX2-8* mutations in NTDs patients.

Using the tractable genome of the dog for association mapping of naturally occurring NTDs, we identified a frameshift mutation in *NKX2-8*. Additionally, rare missense variants in *NKX2-8* were identified in 4% of the cases in a cohort of spina bifida patients. To the best of our knowledge, this is the first documentation of a potential role for *NKX2-8* in the development of NTDs. Future functional studies are required in order to provide insights into the mechanisms and etiologies which constitute NTDs in both species.

## Materials and Methods

### Ethics statement

All research involving human participants was approved by Northwestern University (Chicago) and the University of Iowa institutional review boards (IRBs). Informed consent was obtained and all clinical investigation must have been conducted according to the principles expressed in the Declaration of Helsinki.

DNA samples of domestic dogs (Canis familiaris) owned by private individuals were used in this study. We accepted samples from dogs of all ages and of both sexes. The sample collection protocol was approved by the University of California, Davis Animal Care and Use Committee (protocol #16892).

### Sample collection

Weimaraner samples were solicited using advertisements posted in the Weimaraner Club of America (WCA) magazine, on the WCA website, by direct communication with Weimaraner owners and treating veterinarians, and via the Veterinary Information Network (VIN). All of the samples used in this study came from shorthaired American dogs. Presumed (no histopathology) spinal dysraphism cases were brought to our attention by treating clinicians, Weimaraner breeders and owners. Veterinary evaluation of congenital non-progressive neurological abnormalities which consisted of pelvic limb ataxia, paraparesis, and delayed proprioceptive positioning in the pelvic limbs; together with patient signalment and history served to make a suggestive diagnosis. Samples from additional breeds of dogs were obtained from patients of the Veterinary Medical Teaching Hospital at UC Davis.

### DNA extraction

DNA was extracted from blood samples in EDTA using a commercially available kit (Puregene, Gentra Systems, Minneapolis, MN). Additional DNA isolation from buccal swabs was performed as previously described [54].

### Genome-wide association study

DNA samples were genotyped using the Illumina 170K CanineHD BeadChip (Illumina, San Diego, CA). Quality control checks on the canine dataset were performed for individuals and SNPs using GenABEL in the R statistical package [55]. SNPs were excluded if they had a minor allele frequency (MAF)<5%, a genotype call rate <95%, or if they deviated from the Hardy-Weinberg Equilibrium (HWE). A total of 114,775 SNPs passed the quality control check and were available for analysis. The retained SNPs were then used for case-control chi-square statistical analysis by PLINK [56], and Manhattan and quantile-quantile (QQ) plots were generated using GenABEL [55]. We assessed the effect of population stratification by examining the QQ plots for deviation of the p-values from the null hypothesis. We considered a significant genome-wide association if the SNPs p-value was below the 5% Bonferroni-corrected threshold (p≤0.05; −log 10≥1.3). To derive the genome-wide significance thresholds we repeated the GWAS with 100K Max (T) permutations.

### RNA/cDNA preparation

Adult beagle total RNA was obtained from Zyagen (San Diego, CA, USA). cDNA was synthesized with the SuperScript III First-Strand Synthesis System for RT-PCR (life technologies, Grand Island, NY 14072, USA). Primers for the complete cDNA of *NKX2-8* were designed using the Primer3 program [57]. cDNA PCR products were cloned using the TOPO TA Cloning kit (pCR2.1-TOPO vector) with One Shot TOP10 Chemically Competent E. coli (life technologies, Grand Island, NY 14072, USA). Products were isolated with the Qiaprep Spin Miniprep kit (QIAGEN, Valencia, CA 91355, USA) and sequenced as described below. Nucleotide sequences were translated into amino-acid sequences with Vector NTI software (Applied Biosystems, CA 92008).

### Primers

Primers for the two exons and for the 3 and 5′ UTRs of *NKX2-8* were designed using the Primer3 program [57] (Table S3). The primers, E2F2: 5′ CTGGTAGGCGGGGAAGAG; and E2R2: 5′ GGTTCCAGAACCATCGCTAC, were used to generate PCR products which flank the frameshift mutation within exon 2 of the *NKX2-8* gene. PCR was performed using 40 ng of DNA, 1 unit of AccuPrime GC-Rich DNA Polymerase, 5 ul of buffer A (AccuPrime high GC DNA polymerase kit; life technologies, Grand Island, NY 14072, USA), 50 ng forward and reverse primers, in 25 ul reaction volume. Cycle conditions of 3 min at 95°C followed by 35 cycles of 30 s at 95°C, 30 s at 62°C, and 1 min at 72°, with a final extension of 20 min at 72°C were used. Primers (Table S1) were used to generate overlapping sequences to complete the genomic sequence of *NKX2-8*. The sequence upstream of the gene was missing on the May 2005 CanFAm2.0 genome assembly (viewed using the UCSC genome browser), and was captured using the LongAmp *Taq* PCR Kit (New England BioLabs Ipswich, MA 01938, USA).

The PCR products were electrophoresed on 1–2% agarose, and cleaned using ExoSAP-IT. Purified PCR products were sequenced using the Big Dye terminator mix on ABI 3500 Genetic Analyzer (Applied Biosystems, CA 92008). Sequences were visualized using Chromas2 (Technelysium, Tewantin, QLD, Australia) and analyzed with Vector NTI software (Applied Biosystems, CA 92008).

### Human patient cohorts

Cases comprised a total of 149 patients. Unrelated European American (n = 130), and unrelated African American (n = 19) samples from patients with lumbosacral myelomeningocele (spina bifida). Collected at Children's Memorial Hosptial in Chicago, IL, USA. All cases had open spina bifida (myelomeningocele).

### Sequencing of human samples

Genomic DNA fragments spanning the two exons of *NKX2-8* were amplified by PCR. Purified PCR products were sequenced using Big Dye terminator chemistry (Applied Biosystems) and analyzed on a MegaBACE 1000 (Amersham). Sequence reads derived from both strands were assembled, aligned and analyzed for nucleotide differences using Sequencher (GeneCodes). We assessed the presence of variants in the Exome Variant Server, NHLBI GO Exome Sequencing Project (ESP), Seattle, WA (URL: http://evs.gs.washington.edu/EVS/); data release ESP6500, November 2012. PCR primers and conditions used are shown in Table S4.

NCBI BLASTP [58] was used to compare protein sequence conservation across species. The biological sequence alignment tool, Bio Edit, (Ibis Biosciences, Carlsbad, CA), was used to align protein sequences. The PolyPhen online tool was used to predict the possible impact of missesne mutations [59].

## Supporting Information

Figure S1 Abnormal posture in a female Weimaraner with spinal dysraphism (case #2), photographed at 2 years of age. **A**. The pelvic limbs are positioned in an abnormally extended posture, with the right pelvic limb knuckled over. **B**. The pelvic limbs are positioned abnormally (too far to the left and underneath the dog).
(TIF)

Figure S2 Quantile–Quantile plots of the GWAS results. **A**. deviation from the null line (p-values between zero and one) of inflated p-values suggestive of an association (λ = 1.03) **B**. After removal of the SNPs on chromosome 8, the distribution of the p-values fits the null hypothesis (λ = 1.01).
(TIF)

Table S1 Regional candidate genes on canine Chromosome 8 (position 17,681,036–19,114,924)
(DOCX)

Table S2 Allele frequencies of altered amino acids within *NKX2-8* in cases of spina bifida compared to controls
(DOCX)

Table S3 Primers used to generate overlapping segments of the *NKX2-8* gene from genomic DNA.
(DOCX)

Table S4 Primers used to sequence the exons of *NKX2-8* in human patients
(DOCX)

Video S1 Abnormal pelvic limb gait in a female Weimaraner with spinal dysraphism (case #2) documented at 3 months of age, and a normal adult female Weimaraner. The puppy's gait is typical for a dog with spinal dysraphism. Both pelvic limbs move together simultaneously (so called “bunny hopping”). The puppy is also ataxic and paraparetic.
(WMV)
