# Supplementary figures and images for: Genome-Wide Association Mapping in Dogs Enables Identification of the Homeobox Gene, NKX2-8, as a Genetic Component of Neural Tube Defects in Humans
Source: PLoS Genet. 2013 Jul 18;9(7):e1003646. doi: 10.1371/journal.pgen.1003646 (PMC3715436; doi:10.1371/journal.pgen.1003646)

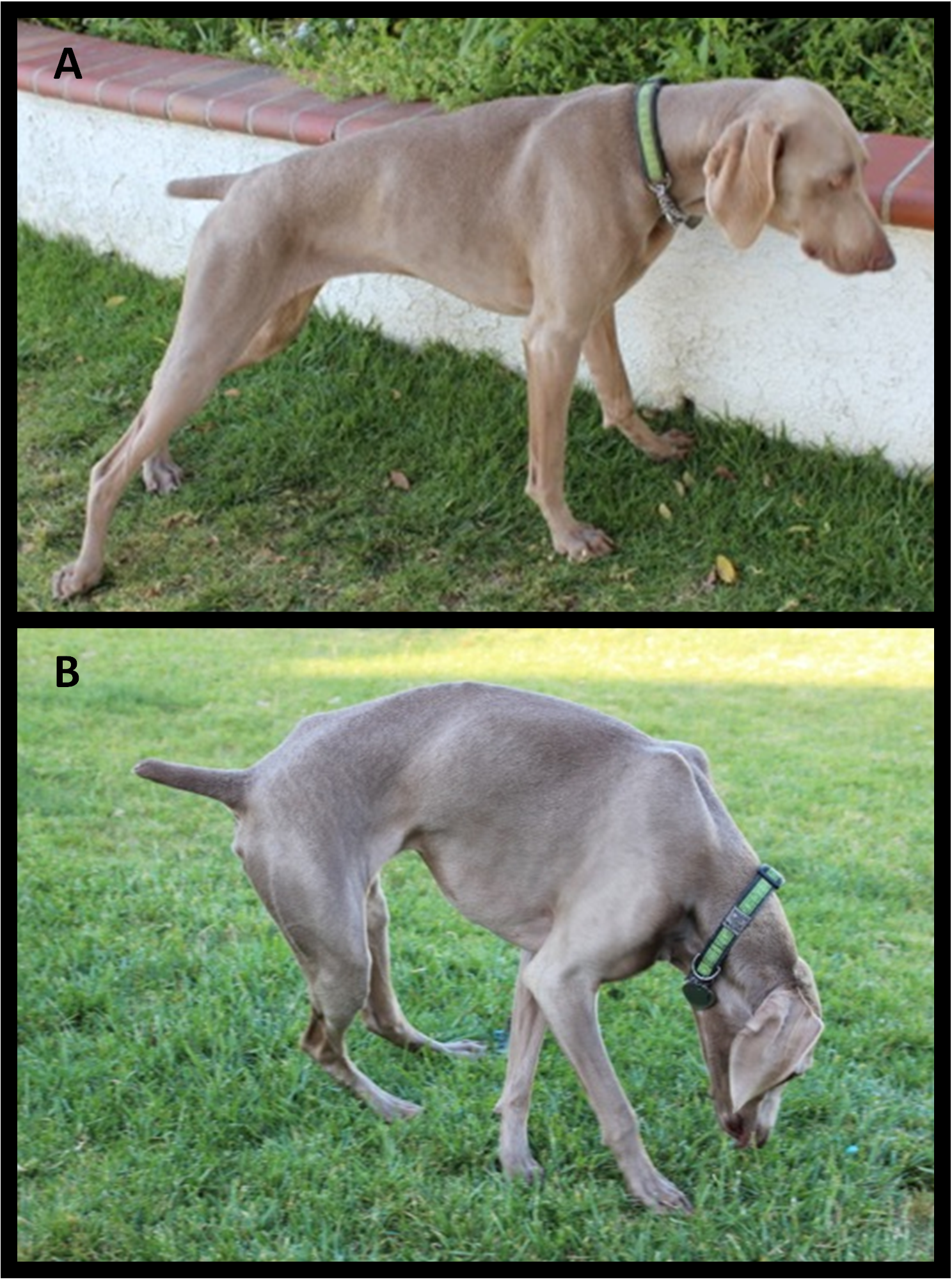

Supplement: Figure S1 — Abnormal posture in a female Weimaraner with spinal dysraphism (case #2), photographed at 2 years of age. A. The pelvic limbs are positioned in an abnormally extended posture, with the right pelvic limb knuckled over. B. The pelvic limbs are positioned abnormally (too far to the left and underneath the dog). (TIF) [file pgen.1003646.s001.tif]

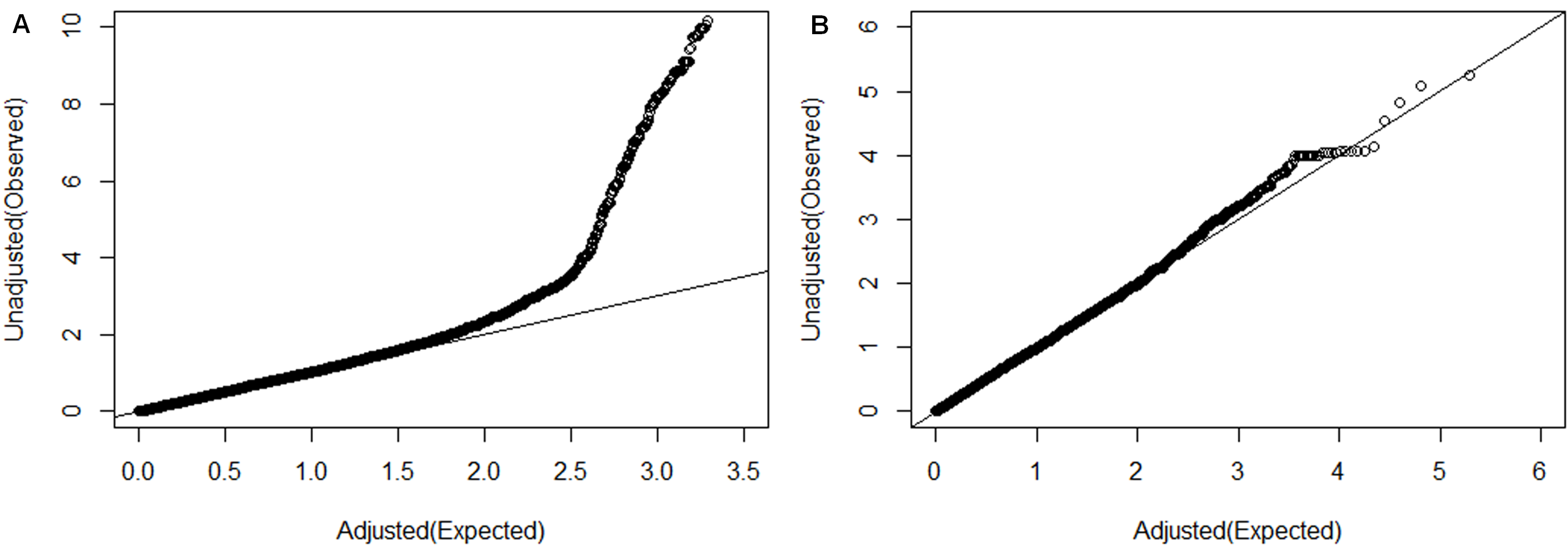

Supplement: Figure S2 — Quantile–Quantile plots of the GWAS results. A. deviation from the null line (p-values between zero and one) of inflated p-values suggestive of an association (λ = 1.03) B. After removal of the SNPs on chromosome 8, the distribution of the p-values fits the null hypothesis (λ = 1.01). (TIF) [file pgen.1003646.s002.tif]
