# Supplementary material for: Genome-Wide Association Mapping in Dogs Enables Identification of the Homeobox Gene, NKX2-8, as a Genetic Component of Neural Tube Defects in Humans
Source: PLoS Genet. 2013 Jul 18;9(7):e1003646. doi: 10.1371/journal.pgen.1003646 (PMC3715436; doi:10.1371/journal.pgen.1003646)
Supplement: Table S1 — Regional candidate genes on canine Chromosome 8 (position 17,681,036–19,114,924) (DOCX) [file pgen.1003646.s003.docx]

| **CHR 8 location** | **Gene** | **Function** |
| --- | --- | --- |
| 17,703,816-17,704,142 | RPL36AL | Ribosomal protein |
| 17,890,282-17,912,716 | MBIP | MAP3K12 binding inhibitory protein |
| 18,093,602-18,094,339 | TITF1 | Homeobox gene expressed in thyroid |
| 18,145,579-18,145,719 | RPL39L | Ribosomal protein |
| 18,156,525-18,157,928 | Nkx2-8 | Homeobox gene expressed in developing neural tube |
| 18,229,798-18,243,549 | PAX9 | Paired box gene expressed in teeth |
| 18,247,726-18,430,946 | SLC25A21 | Mitochondrial protein |
| 18,333,836-18,334,180 | AK127847 | Expressed in placenta |
| 18,552,268-18,553,499 | PIP5K2A | Kinase protein |
| 18,589,730-18,590,002 | SNRPE | Small nuclear ribonucleoprotein E |
| 18,730,148-18,730,480 | MGC70863 | Mitochondrial protein |
| 18,730,154-18,730,579 | RPL23A | Ribosomal protein |
| 18,730,223-18,730,603 | BC096706 | Ribosomal protein |
| 18,748,452-18,749,534 | AK097143 | Expressed in placenta |
| 18,800,554-19,043,003 | MIPOL1 | Mirror-image polydactyly gene 1 protein |
| 18,800,554-19,000,545 | BC036124 | Mirror-image polydactyly gene 1 protein |
| 18,864,366-18,864,822 | RPS11 | Ribosomal protein |
| 19,085,869-19,089,654 | FOXA1 | Transcription factor, multiple organs |

Table S1: Regional candidate genes on canine Chromosome 8 (position 17,681,036-19,114,924)
