# Supplementary material for: Genome-Wide Association Mapping in Dogs Enables Identification of the Homeobox Gene, NKX2-8, as a Genetic Component of Neural Tube Defects in Humans
Source: PLoS Genet. 2013 Jul 18;9(7):e1003646. doi: 10.1371/journal.pgen.1003646 (PMC3715436; doi:10.1371/journal.pgen.1003646)
Supplement: Table S2 — Allele frequencies of altered amino acids within NKX2-8 in cases of spina bifida compared to controls (DOCX) [file pgen.1003646.s004.docx]

Table S2: Allele frequencies of altered amino acids within *NKX2-8* in cases of spina bifida compared to controls

|  | **Change of amino acid** | **No change of amino acid** | **Total** |
| --- | --- | --- | --- |
| **Spina bifida samples** | 5 | 255 | 260 |
| **Exome Variant Server** | 72 | 8,428 | 8500 |
| **Total** | 77 | 8,683 | 8,760 |
| One tailed Chi-squared analysis with Yate’s correction (p=0.036) | | | |
