# Supplementary material for: Genome-Wide Association Mapping in Dogs Enables Identification of the Homeobox Gene, NKX2-8, as a Genetic Component of Neural Tube Defects in Humans
Source: PLoS Genet. 2013 Jul 18;9(7):e1003646. doi: 10.1371/journal.pgen.1003646 (PMC3715436; doi:10.1371/journal.pgen.1003646)
Supplement: Table S3 — Primers used to generate overlapping segments of the NKX2-8 gene from genomic DNA. (DOCX) [file pgen.1003646.s005.docx]

| Primer | *NKX2-8* location | Primer Sequence | Ta (◦c) | Product Size (primers) bp | Comments |
| --- | --- | --- | --- | --- | --- |
| E2F1 | exon 2 | 5’ CCGGCACTCATTCAGGTACT | 60.1 |  |  |
| E2R1 | exon 2 | 5’ CGGTCTTCCGCTTACTCACA | 61.3 | (E2F1-E2R1) 398 |  |
| E1F1 | exon 1 | 5’ AGAGCCTCTGTCCTCTGGTC | 58.6 |  |  |
| E1F2 | exon 1 | 5’ AGCATTTAAGGGCCGTTTCT | 60.1 |  |  |
| E1R2 | exon 1 | 5’ CTCGCCACCCAAGATGTTAT | 60.0 | (E1F1-E1R2) 823 |  |
| gapF3 | 3’UTR | 5’ TCAAGCACATCTCCTTGCAG | 60.1 |  |  |
| gapR3 | 3’UTR | 5’ GGTGTCCTGGAACTGGTGAG | 60.6 | (gapF3-gapR3) 1297 | LongAmp® *Taq* |
| cDNA_F | 3’UTR | 5’ GGGGGATATCCTGGCTAAAG | 59.8 |  |  |
| cDNA_R | 5’UTR | 5’ CTGTAAGCGCGCTAATATCC | 57.7 |  |  |
| E1F3 | exon 1 | 5’ CCGCTTACTCACAGGGGTAG | 59.8 | (E1F3-E1R2) 756 |  |
| E1R3 | exon 1 | 5’ CTCTGGGCGCCTCAGTTT | 61.5 | (E1F1-E1R3) 306 ;  ( E1F3-E1R3) 199 |  |
| E2F2 | exon 2 | 5’ CTGGTAGGCGGGGAAGAG | 60.7 |  |  |
| E2R2 | exon 2 | 5’ GGTTCCAGAACCATCGCTAC | 59.6 | (E2F2-E2R2) 290 | used for genotyping |

**Table S3**: Primers used to generate overlapping segments of the *NKX2-8* gene from genomic DNA.
