# Supplementary material for: Genome-Wide Association Mapping in Dogs Enables Identification of the Homeobox Gene, NKX2-8, as a Genetic Component of Neural Tube Defects in Humans
Source: PLoS Genet. 2013 Jul 18;9(7):e1003646. doi: 10.1371/journal.pgen.1003646 (PMC3715436; doi:10.1371/journal.pgen.1003646)
Supplement: Table S4 — Primers used to sequence the exons of NKX2-8 in human patients (DOCX) [file pgen.1003646.s006.docx]

| ***NKX2-8* exon** | **Direction** | **Primer sequence** | **Annealing temperature (**◦c) |
| --- | --- | --- | --- |
| Exon1 | forward | CAGAGATTCCGCTGTAAATGC | 56 |
|  | reverse | CTGGGATTTCGGCTTTCC |  |
| Exon2A | forward | CCGAAAGTCTCGGGCTAGT | 56 |
|  | reverse | GCTTCAGCTTGTAGCGATGA |  |
| Exon2B | forward | GCAGCAGCGGTACCTGTC | 55 |
|  | reverse | CTGCTCCAATCGCAGAGC |  |

**Table S4**: Primers used to sequence the exons of *NKX2-8* in human patients
